# Influences of temperature and moisture on abiotic and biotic soil CO_2_ emission from a subtropical forest

**DOI:** 10.1186/s13021-021-00181-8

**Published:** 2021-05-25

**Authors:** Xiaomei Chen, Muying Liu, Zhanying Xu, Hui Wei

**Affiliations:** 1 School of Geography and Remote Sensing, Guangzhou University, Guangzhou, 510006 China; 2 College of Natural Resources and Environment, South China Agricultural University, Guangzhou, 510483 China; 3 Guangdong Provincial Key Laboratory of Eco-circular Agriculture and Key Laboratory of Agro-Environment in the Tropics, Ministry of Agriculture and Rural Affairs, South China Agricultural University, Guangzhou, 510642 China

**Keywords:** Soil organic carbon decomposition, Soil respiration, Temperature sensitivity, Moisture sensitivity, Biotic and abiotic factors

## Abstract

**Background:**

Soil CO_2_ efflux is considered to mainly derive from biotic activities, while potential contribution of abiotic processes has been mostly neglected especially in productive ecosystems with highly active soil biota. We collected a subtropical forest soil to sterilize for incubation under different temperature (20 and 30 °C) and moisture regimes (30%, 60 and 90% of water holding capacity), aiming to quantify contribution of abiotic and biotic soil CO_2_ emission under changing environment scenarios.

**Main findings:**

Results showed that abiotic processes accounted for a considerable proportion (15.6−60.0%) of CO_2_ emission in such a biologically active soil under different temperature and moisture conditions, and the abiotic soil CO_2_ emission was very likely to derive from degradation of soil organic carbon via thermal degradation and oxidation of reactive oxygen species. Furthermore, compared with biotically driving decomposition processes, abiotic soil CO_2_ emission was less sensitive to changes in temperature and moisture, causing reductions in proportion of the abiotic to total soil CO_2_ emission as temperature and moisture increased.

**Conclusions:**

These observations highlight that abiotic soil CO_2_ emission is unneglectable even in productive ecosystems with high biological activities, and different responses of the abiotic and biotic processes to environmental changes could increase the uncertainty in predicting carbon cycling.

## Background

Soil constitutes to the greatest terrestrial carbon (C) pool, with a size > 2000 Pg organic C in upper 2 m depth of land [1], which is around three times the atmospheric C pool. Soil CO_2_ emission, often termed as soil respiration, may substantially affect CO_2_ concentration in the atmosphere and then the global climate system [2], since it is the largest terrestrial C source to the atmosphere [3]. Soil respiration is generally separated into two components, i.e., autotrophic and heterotrophic respirations, and on global scale the latter contributes an increasing proportion of the total soil respiration in recent decades [4, 5]. The global heterotrophic respiration is estimated to be 50.3 ± 25.0 Pg C yr^− 1^ during 1982–2012, a figure that is much higher than its autotrophic counterpart (35.2 Pg C yr^− 1^) [6], therefore highlighting a necessity to deepen our understanding on the sources and variations of soil heterotrophic respiration.

Soil organic C (SOC) decomposition is traditionally regarded to derive mainly from biotic activities (especially microbial activities, R_biotic_) and be pooled as heterotrophic respiration in most of previous studies, although it is widely accepted that soil CO_2_ may be produced by both the biotic and abiotic processes. In addition to microbial driven SOC decomposition, diverse abiotic pathways, such as photodegradation, thermal degradation, oxidation of reactive oxidative species (ROSs), extracellular oxidative metabolism, and inorganic chemistry reactions, may also contribute to CO_2_ emission from the soil (R_soil_) and therefore produce soil abiotic CO_2_ emission (R_abiotic_) [7, 8]. These abiotic C decomposition pathways have been recognized especially in litter decomposition studies, because a considerable number of studies have reported photodegradation of litter under different conditions [9–11]. However, soil R_abiotic_ has been scarcely quantified, except few studies conducted in harsh environments such as arid and polar soils where biotic activities are extremely low [12, 13]; the abiotic processes may contribute up to 99.5% of the total soil CO_2_ emission, e.g., in an alkaline soil of the southern Gurbantunggut Desert region, China [12]. In those biologically active ecosystems, contribution of the abiotic to total soil CO_2_ emission is assumed tiny and often overlooked, although recent studies highlighted the importance of nonmicrobial CO_2_ emission and possible production pathways in the soil [7, 8].

Environmental factors including temperature and moisture regimes may play critical roles to regulate the rate of soil CO_2_ emission. A consensus has been almost reached that temperature can exponentially affect soil CO_2_ efflux rate, and the global average temperature sensitivity is estimated to be 3.0 ± 1.1 over air temperature ranging 0–20 ℃ [14]. Soil moisture could significantly affect soil CO_2_ emission, especially the heterotrophic component [15, 16]. These are attributable to the fact that biotic activities are to a great extent supported by supplies of energy and water. However, chemical reactions will also be accelerated by warming temperature, as Arrhenius theory demonstrates, and soil moisture could potentially influence abiotic SOC decomposition by adjusting supplies of substrates and oxygen [15]. Therefore, changes in environmental conditions probably induce variations in the R_abiotic_. Nevertheless, this topic to the best of our knowledge is still virgin, despite we are facing scenarios of global warming and precipitation changes.

In this study, we sterilized a productive soil with high biotic activities for incubation to investigate the contribution of abiotic and biotic soil CO_2_ emission, and different regimes of incubation temperature (20 and 30 °C) and moisture (30%, 60 and 90% of water holding capacity [WHC]) were set up as experimental factors. The soil microbial biomass and activity of the studied soil are high as observed in previous studies [17, 18]. This experiment was conducted with aims: (1) to quantify what were the proportions of R_abiotic_ and R_biotic_ to the total soil CO_2_ emission, in order to verify whether abiotic soil CO_2_ emission is neglectable in ‘biologically active’ soils; and (2) to investigate how the rate and proportion of R_abiotic_ and R_biotic_ would vary under changing temperature and moisture scenarios.

## Results and discussion

**Fig. 1:**
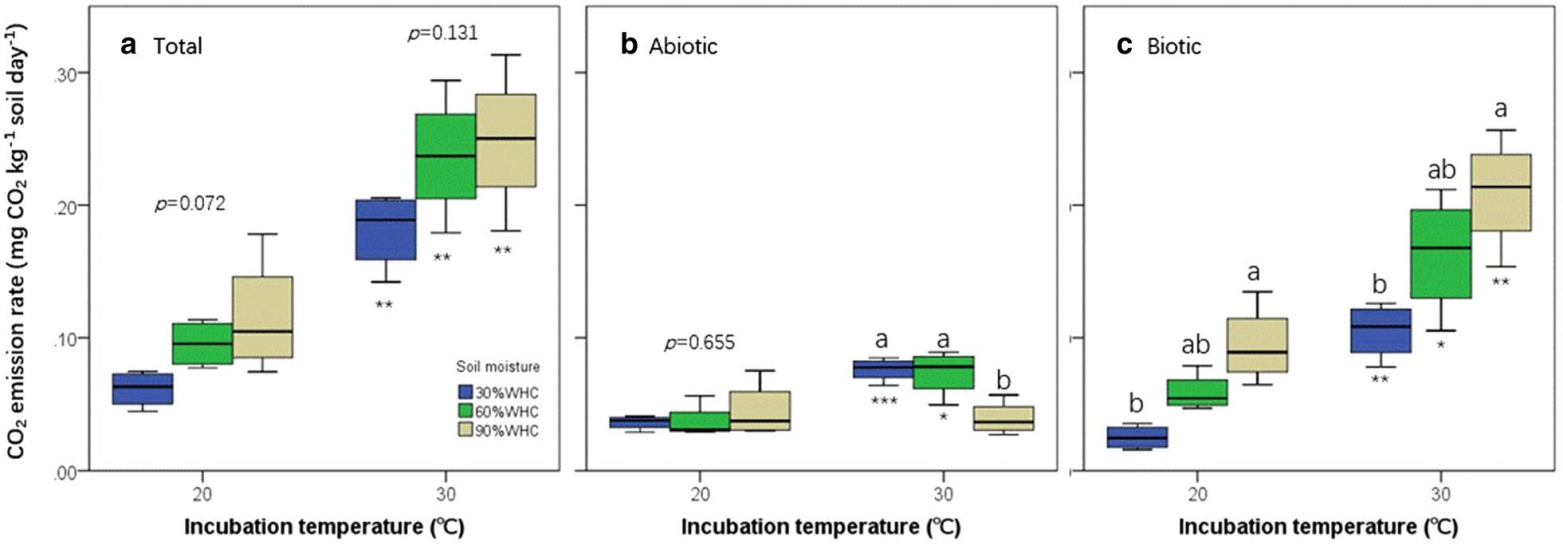
Total, abiotic and biotic soil CO_2_ efflux rate under different temperature and moisture regimes. In each panel, different lowercase letters indicate significant differences among moisture regimes, and asterisks (*) indicate significant differences between the soil CO_2_ efflux rate between high and low temperatures

We observed that R_soil_ ranged from 0.061 ± 0.0069 (under 20 ℃ & 30% WHC) to 0.25 ± 0.027 mg kg^− 1^ soil day^− 1^ (under 30 ℃ & 90% WHC) under different combinations of temperature and moisture regimes, and increasing temperature or moisture significantly or tended to promote R_soil_ in this study (Fig. 1A). It is not surprising because such positive relationships in a certain temperature or moisture range have been frequently reported for soil CO_2_ emission, although the range may be variable depending on soil type and properties [15, 16, 19].

Interestingly, both the R_abiotic_ and R_biotic_ contributed obviously to R_soil_ under each treatment (Fig. 1; Table 1). Under 20 °C, R_abiotic_ was 0.036 ± 0.0027, 0.037 ± 0.0065 and 0.045 ± 0.011 mg kg^− 1^ soil day^− 1^ at 30%, 60 and 90% of WHC, respectively, and the rate at 30 °C was 0.076 ± 0.0044, 0.074 ± 0.0087 and 0.039 ± 0.0045 mg kg^− 1^ soil day^− 1^ correspondingly (Fig. 1B, C). This produces a considerable proportion (on average 36.7%) of R_abiotic_ to R_soil_ in the studied soil (Table 1), highlighting a necessity to investigate abiotic soil CO_2_ emission even in those fertile soils with relatively high biotic activities. This observation may be supported by the result that the studied soil contains a considerable proportion of chemical readily-oxidizable SOC that may be easily degraded by abiotic processes [20, 21].

**Table 1:** Proportion of abiotic and biotic to total CO_2_ efflux rate in the subtropical forest soil

	Abiotic			Biotic		
	20 °C	30 °C	|*t*|	20 °C	30 °C	|*t|*
30 %WHC	60.0 ± 3.0 % a	42.3 ± 1.3 % a	29.2**	40.0 ± 3.0 % b	57.7 ± 1.3 % b	29.2**
60 %WHC	38.4 ± 4.6 % b	31.8 ± 4.4 % a	1.1	61.6 ± 4.6 % a	68.2 ± 4.4 % b	1.1
90 %WHC	31.9 ± 4.5 % b	15.6 ± 1.0 % b	12.5*	68.1 ± 4.5 % a	84.4 ± 1.0 % a	12.5*
*F*	12.8**	24.4***		12.8**	24.4***	

Water holding capacity is abbreviated as WHC. Data are presented as mean ± standard error (n = 4). Statistical *F* and *t* values are shown, with asterisks *, **, and *** indicating significant differences at *p* < 0.05, 0.01 and 0.001, respectively. In each column, different lowercase letters indicate significant differences at *p* < 0.05 among different soil moisture treatments

Abiotic soil CO_2_ efflux may derive from solar-induced organic matter degradation (i.e., photodegradation of the functional group of carboxyl and glucose), thermal degradation by dissociating chemical bonds of the SOC, oxidation of ROSs that could be produced by biotic or photochemical processes, extracellular oxidative metabolism, and inorganic chemistry reactions [8]. Photodegradation and inorganic chemistry reactions have been reported, though limited, to contribute to the total soil CO_2_ emission in previous studies [9, 12, 13]. In this study, however, the mechanisms of photodegradation, extracellular oxidative metabolism and inorganic chemical reactions may not contribute obviously to the observed high proportion of R_abiotic_, due to (i) dark incubation used, (ii) high temperature (121 ℃ in the process of sterilization) resulting in inactivation of exoenzymes, (iii) low carbonate content in such a strongly acidic soil (pH_water_ = 3.6 ± 0.07).

Thermal degradation and ROSs oxidization could mainly account for the R_abiotic_ component of the tested soil in the present study. Regarding thermal degradation of SOC, most studies have employed high temperatures that are well above the ignition temperature of organic materials (often higher than 140 °C) [22, 23], and therefore it seems that thermal degradation might not have functioned under relatively low temperatures, e.g., 20 and 30 °C as used in this study. However, previous studies have reported obvious CO_2_ emission due to thermal degradation of organic materials derived from plant residues under relatively low temperature conditions, and the CO_2_ emission rate exponentially increased with the increase of incubation temperature ranging from 25 to 55 °C [24]. This suggests that SOC, of which a considerable portion derives from plant residues and excretions, may also be thermally degraded under relatively low temperatures below the ignition point of organic materials, although we could not quantify the exact proportion of contribution of thermal degradation to the total soil CO_2_ emission. Similarly, we also observed obviously higher abiotic soil CO_2_ emission rate under 30 °C than under 20 °C, especially at the water regimes of 30 and 60% WHC (Fig. 1B).

Besides the thermal degradation pathway, the ROSs oxidation pathway could also contribute substantially to the soil abiotic CO_2_ emission. As previous studies demonstrated, existence of iron (Fe) and magnesium (Mn) may substantially contribute to soil CO_2_ emission via producing ROSs (such as hydroxyl radical and hydrogen peroxide) to oxidize SOC [25–27]. The studied soil is a type of highly weathered soil that is rich in the Fe content and contains detectable Mn content [28]. These soil compounds, probably some others not analyzed in our studies, can produce a considerable amount of ROSs in oxidation-reduction reactions [25, 29], consequently contributing to the abiotic soil CO_2_ emission as observed in this study (Fig. 1). Despite the abiotic oxidation potential of Fe and Mn, we also recognize that existence of soil organisms may substantially accelerate the Fe and Mn mediated abiotic oxidation process, since production of Fe (II) and Mn (III) are predominantly regulated by soil microorganisms [27].

In this study, R_biotic_ was significantly higher under high than low temperatures (Fig. 1C), consistently among the soil moisture treatments (interactive effects of two-way ANOVAs: *F* = 0.707, *df* = 2, *p* = 0.507), since metabolic reactions in soil organisms could be accelerated as temperature increased [30]. This is not controversial. Unlike on biotic components, however, temperature effects on R_abiotic_ were not consistent under different soil moisture conditions, as indicated by the significant interactive effect between incubation temperature and moisture treatments (interactive effects of two-way ANOVAs: *F* = 6.577, *df* = 2, *p* = 0.007). Under 30 and 60% of WHC, R_abiotic_ was also significantly higher under high than low temperatures, whereas it was comparable between the high and low temperature treatments of this study under 90% WHC treatment (Fig. 1B). This might be associated with limited movement and supply of oxygen under the water-rich soil where water could occupy most space in the soil aggregates [15]. In this condition, chemical reaction rate could not be promoted with increasing temperature due to lack of oxygen as a necessary reactant. Further studies to partition contribution of the abiotic processes would be helpful to clearly understand such inconsistent temperature effects on R_abiotic_ among varying soil moisture conditions.

**Fig. 2:**
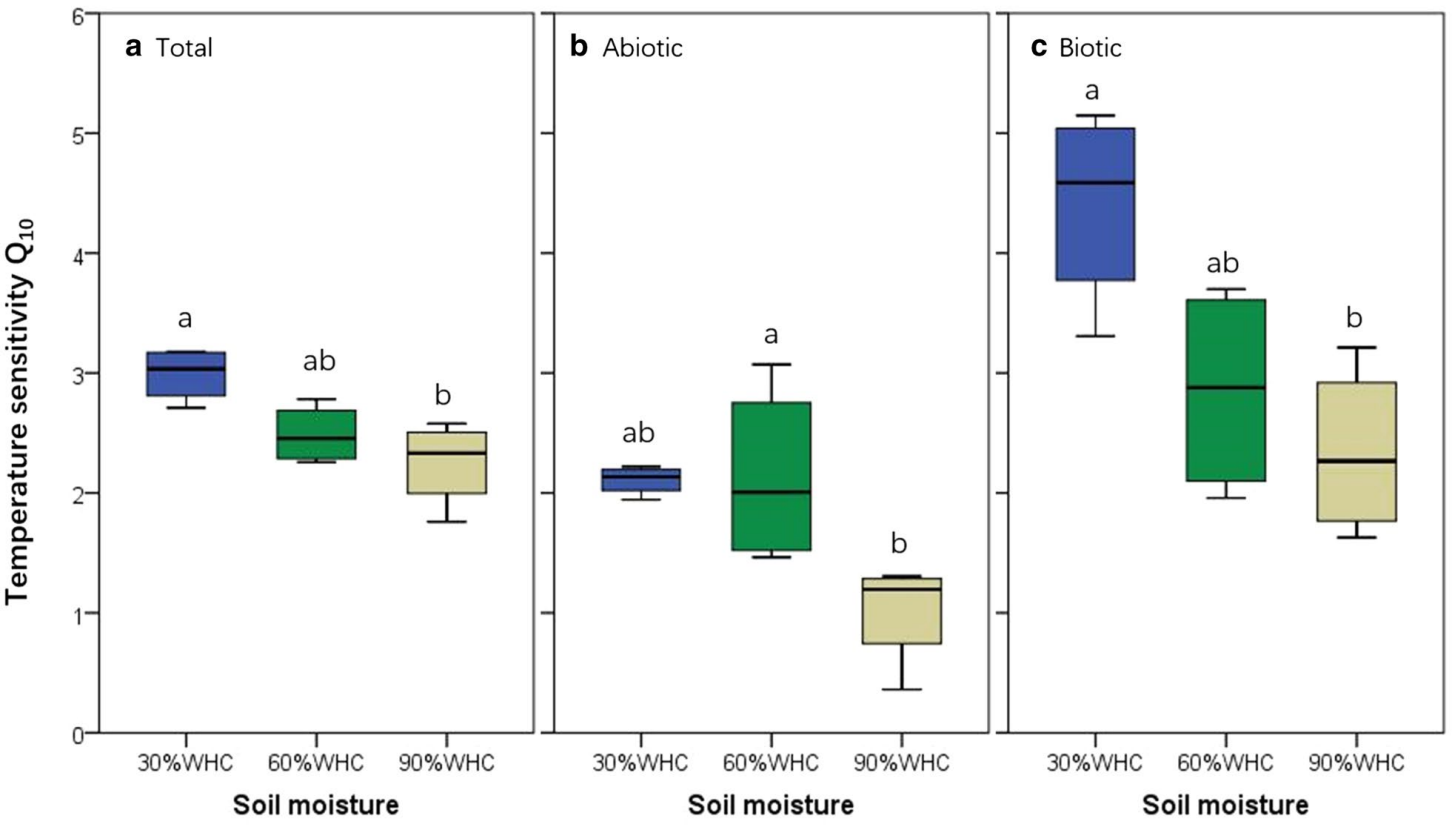
Temperature sensitivity (Q_10_) of the total, abiotic and biotic soil CO_2_ efflux rate at 30%, 60 or 90% of water holding capacity (WHC). In each panel, different lowercase letters indicate significant differences among moisture regimes

Similar with rate changes, the proportion of R_biotic_ to R_soil_ significantly increased with temperature increases (Table 1). For R_abiotic_, however, its proportion significantly or tended to decrease as temperature increased (Table 1), despite warming temperature could promote it under 30 and 60% of WHC conditions (Fig. 1B). This result implies that biological processes are more sensitive to warming than abiotic processes, which is also evidenced by the greater Q_10_ (rate of change of soil CO_2_ emission with temperature increases by 10 °C) of R_biotic_ than R_abiotic_ (Fig. 2). Therefore, compared with abiotic processes such as solar-induced and thermal degradation, C emission related metabolic activities may be more promoted by global warming. Considering abiotic soil CO_2_ emission and its response to temperature changes could result in lower positive feedbacks of soil C emission to temperature warming than that projected previously while the abiotic soil CO_2_ emission was not considered.

Noteworthily, soil moisture affected R_abiotic_ and R_biotic_ in inverse ways (Fig. 1; Table 1). Increases in soil moisture did not significantly alter or even significantly reduced (under 30 ℃ & 90%WHC) R_abiotic_ within the investigation period (Fig. 1B). The reduction in R_abiotic_ may be attributable to the fact that soil abiotic CO_2_ emission driven by the ROSs pathway has been affected by changes in soil moisture via regulating soil oxygen availability [31, 32]. Contrastingly, increasing soil moisture significantly increased R_biotic_, which is consistent under the two incubation temperatures (Fig. 1C). As a result, the proportion of R_biotic_ to R_soil_ was significantly higher under high soil moisture conditions and that of R_abiotic_ significantly decreased with the increasing soil moisture, regardless of incubation temperatures (Table 1). Abiotic processes contributed greatest proportion to soil CO_2_ emission under low temperature and low moisture conditions (i.e., 20 °C & 30% WHC in this study), under which R_biotic_ was low (Fig. 1A). Likewise in a previous study, Ma et al. found that abiotic components dominated soil CO_2_ efflux on an alluvial plain in a temperate arid desert where biological activities were weak in the soil [12]. It has been recognized that abiotic decompositions are important for litter decomposition [9, 24], and that abiotic soil CO_2_ emission may dominate soil CO_2_ efflux in harsh environments such as several arid or tundra ecosystems [12, 13]. However, the potential importance of abiotic decomposition of SOC has been largely overlooked in previous studies, especially in biologically active ecosystems. Our result indicates that abiotic processes may contribute a considerable proportion of total soil CO_2_ emission in (sub)tropical forests which have high soil biotic activities. Therefore, more attention is requested to clarify the variations and mechanisms of such abiotic CO_2_ productions and effluxes from the soil.

Moreover, we also calculated the Q_10_ index to indicate apparent temperature sensitivity of soil CO_2_ emission [30]. Within the temperature range between 20 and 30 ℃, Q_10_ of R_soil_ was 2.57 ± 0.12, a value that is a bit lower than the global average Q_10_ (3.0 ± 1.1) of soil CO_2_ efflux (including autotrophic respiration) over 0–20 ℃ range of air temperature [14]. This may be associated with relatively higher temperature sensitivity of autotrophic than heterotrophic respiration [33] or/and thermal acclimation of SOC decomposition, i.e., higher temperature sensitivity under low than high temperatures [19, 30, 34]. Air temperature in the study site had been increasing by 1.0 ± 0.1 ℃ during past five decades (1954–2009) [35] and the lasting increase in temperature may have consequently reduced the magnitude of C sink [36], due to extra soil C loss induced by warming [37].

Furthermore, Q_10_ of R_abiotic_ and R_biotic_ were 1.75 ± 0.21 and 3.20 ± 0.34, respectively (Fig. 2B, C). Such a high Q_10_ of R_biotic_, compared with R_soil_ and R_abiotic_, indicates high sensitivity of biotic decomposition activity to warming and therefore raising temperature could increase the proportion of biotic to total SOC decomposition (Table 1). Under high soil moisture conditions (such as 90% WHC in this study), however, Q_10_ (1.01 ± 0.12) of R_abiotic_ was not significantly different from one (One-Sample *t* = 0.065, *df* = 3, *p* = 0.952), suggesting R_abiotic_ to be independent of temperature increases in water-rich soil conditions. Moreover, high soil moisture (i.e., 90% WHC) could significantly reduce Q_10_ of both R_abiotic_ and R_biotic_, consequently declining Q_10_ of R_soil_. This may be linked to limited oxygen supply under high soil moisture [15], because oxygen availability is important to controlling temperature sensitivity of soil CO_2_ emission [38]. As observed in previous studies, oxygen availability may positively affect not only the microbial driving SOC decomposition [29, 39], but also the abiotic SOC degradation, since the ROSs pathway that produces soil abiotic CO_2_ emission could be regulated by oxygen availability [26, 31]. As a reactant, insufficient oxygen supply under high soil moisture condition could reduce the temperature sensitivity of soil CO_2_ emission and resultantly cause the observed pattern in this study.

Despite the interesting observations, it is notable that two drawbacks exist in the present study. First, this study is a laboratory incubation experiment, in which the intact soil structure and properties may have been substantially disturbed by pretreating processes [40], e.g., sieving and adjusting the soil water content before incubation. This could on the one hand simplify experimental treatments and decrease the heterogeneity of samples and therefore make easier to compare treatment effects [41], which is much useful in such an exploratory study. On the other hand, however, such disturbances are most likely to make observation values away from ‘true values’ [42] and therefore paralleling field observations may be needed to verify the observations. Second, we investigated abiotic soil CO_2_ emission rate after sterilizing soils under high temperature (121 ℃) and biotic component is quantified by subscripting the soil CO_2_ emission rate in the sterilized soil from that in the corresponding unsterilized soil. Despite widely used in related studies, this simplification runs an assumption that the total production of soil CO_2_ is a result of linearly adding up the abiotic and biotic components, which may be not the case under natural conditions since biotic and abiotic C degradation processes are often closely coupled [43]. Therefore, developing advanced methodologies remains needed to improve the estimates of these two components.

## Conclusions

In this study, we observed that abiotic processes contributed a considerable proportion of soil CO_2_ emission in the subtropical forest, with thermal degradation and ROSs oxidation being probably the underlying mechanisms, and abiotic and biotic soil CO_2_ emissions showed different responses to changes in temperature and moisture in this study. Abiotic soil CO_2_ emission has been evidenced to be important in harsh environments such as arid or arctic ecosystems, while it is often considered to be minor and overlooked in ecosystems with high biotic activities. Our observations highlight that the abiotic soil C degradation processes may remain unneglectable even in biologically active ecosystems. As the best of our knowledge, almost all previous modelling studies have overlooked such an important component, which is likely to contribute to the uncertainty of predictions in the ecosystem C cycling under environmental changes. However, the present study has been designed to exploratorily investigate the contribution, as well as temperature and moisture sensitivities, of abiotic and biotic processes to the total soil CO_2_ emission in a biologically active ecosystem, with underlying mechanisms rarely revealed by our observations in this study. It is necessary and much urgent to further explore the mechanisms by which the abiotic soil CO_2_ emission is produced and how it would vary in the changing environmental conditions, especially in the biologically active ecosystems.

## Methods

### Incubation experiment

The studied soil was collected from the surface (0–10 cm) of a subtropical old-growth forest that experienced the subtropical monsoon climate. The forest is the regional climax, with dominate tree species being *Castanopsis chinensis*,*Cryptocarya chinensis*, *Cryptocarya concinna*, and *Erythrophleum fordii etc*. [44]. The soil is lateritic red earth that could be classified as Oxisol according to the US Soil Taxonomy [45]. The soil pH (water extracted) was 3.57 ± 0.073, the SOC content was 36.27 ± 6.07 g kg^− 1^, and the total nitrogen content was 2.00 ± 0.15 g kg^− 1^. The contents of sand, silt and clay are 6.9%, 39.7 and 53.4%, respectively. After collection, the soil samples were immediately transferred with ice bags into lab to sieve for uses.

The sieved soils were separated into two parts. The one was sterilized twice with an interval of 24 h under 121 ℃ to obtain sterile soil, which was incubated to investigate the soil abiotic CO_2_ emission. Two methods, including plate culture and PCR methods, were employed to check the efficiency of soil sterility at the end of incubation experiment and results evidenced no observable soil bacteria grew in the sterilized soils. The other one was not sterilized for incubation to investigate the total soil CO_2_ emission. The difference in the total and abiotic soil CO_2_ emission rate was calculated as soil biotic CO_2_ emission. Both the sterilized and non-sterilized soils were incubated for 64 days under different temperature (20 or 30 ℃) and moisture regimes (30%, 60 or 90% of WHC), with aims to quantify the rate and proportions of R_abiotic_ and R_biotic_ and their response to changes in temperature and moisture conditions in such a biologically active soil. The accumulated CO_2_ concentration released from the soils was determined at days 3, 6, 9, 12, 16, 20, 24, 28, 34, 40, 52, and 64, by using the alkali (NaOH) absorption method.

### Data processes and statistics

The soil CO_2_ efflux rate under each treatment was calculated by fitting the accumulated CO_2_ emission with the incubation period, with the slope of linear regression (for the non-sterilized soil the R^2^s > 0.98, while for the sterilized soil the R^2^s > 0.85) indicating the average efflux rate during the incubation period. The temperature sensitivity index (Q_10_) was calculated by dividing the rate under 30 °C by that under 20 °C [46]. After normality test, the rate of R_abiotic_, R_biotic_ and R_soil_, as well as the proportion of R_abiotic_ or R_biotic_ to R_soil_, were compared between different incubation temperatures by the Paired-Samples T test or among different moisture regimes by the one-way Analysis of Variances (ANOVAs). Two-way ANOVAs were employed to reveal the significance level of interactive effects on the soil CO_2_ emission rate and proportion between incubation temperature and moisture treatments. Tukey Post Hoc Multiple Comparisons were employed to compare the average values between each two groups of the moisture regimes when one-way ANOVAs revealed significant differences. The rate calculations were conducted in R software (version 2.15.2) and all the statistics and figures were finished in IBM SPSS Statistics 22.

## Data Availability

No restrictions are placed on materials, such as materials transfer agreements. Details of all data and materials used in the analysis are available in the main text or on request of the corresponding authors.

## Abbreviations

CO_2_: Carbon dioxide
C: Carbon
Pg: Petagram
yr: Year
SOC: Soil organic carbon
R_bioti_: Soil biotic CO_2_ emission
ROSs: Reactive oxidative species
R_total_: Total soil CO_2_ emission
R_abiotic_: Soil abiotic CO_2_ emission
WHC: Water holding capacity
Fe: Iron
Mn: Magnesium
ANOVA: Analysis of variances
Q_10_: Temperature sensitivity index
PCR: Polymerase chain reaction

## References

[1] Batjes NH (2016). Harmonized soil property values for broad-scale modelling (WISE30sec) with estimates of global soil carbon stocks. Geoderma.

[2] Melillo JM, Frey SD, DeAngelis KM, Werner WJ, Bernard MJ, Bowles FP (2017). Long-term pattern and magnitude of soil carbon feedback to the climate system in a warming world. Science.

[3] Bond-Lamberty B, Thomson A (2010). Temperature-associated increases in the global soil respiration record. Nature.

[4] Bond-Lamberty B, Bailey VL, Chen M, Gough CM, Vargas R (2018). Globally rising soil heterotrophic respiration over recent decades. Nature.

[5] Lei J, Guo X, Zeng Y, Zhou J, Gao Q, Yang Y (2021). Temporal changes in global soil respiration since 1987. Nat Commun.

[6] Lu H, Li S, Ma M, Bastrikov V, Chen X, Ciais P (2021). Comparing machine learning-derived global estimates of soil respiration and its components with those from terrestrial ecosystem models. Environ Res Lett..

[7] Liu J, Hartmann SC, Keppler F, Lai DYF (2019). Simultaneous abiotic production of greenhouse gases (CO_2_, CH_4_, and N_2_O) in subtropical soils. J Geophys Res Biogeosci.

[8] Wang B, Lerdau M, He Y (2017). Widespread production of nonmicrobial greenhouse gases in soils. Global Change Biol.

[9] Rutledge S, Campbell DI, Baldocchi D, Schipper LA (2009). Photodegradation leads to increased carbon dioxide losses from terrestrial organic matter. Global Change Biol.

[10] Austin AT, Mendez MS, Ballare CL (2016). Photodegradation alleviates the lignin bottleneck for carbon turnover in terrestrial ecosystems. Proc Natl Acad Sci United States of Am..

[11] Brandt LA, King JY, Hobbie SE, Milchunas DG, Sinsabaugh RL (2010). The role of photodegradation in surface litter decomposition across a grassland ecosystem precipitation gradient. Ecosystems.

[12] Ma J, Li Y, Liu R (2015). The abiotic contribution to total CO_2_ flux for soils in arid zone. Biogeosci Discussions.

[13] Shanhun FL, Almond PC, Clough TJ, Smith CMS (2012). Abiotic processes dominate CO_2_ fluxes in Antarctic soils. Soil Biol Biochem.

[14] Bond-Lamberty B, Thomson A (2010). A global database of soil respiration data. Biogeosciences.

[15] Yan Z, Bond-Lamberty B, Todd-Brown KE, Bailey VL, Li S, Liu C (2018). A moisture function of soil heterotrophic respiration that incorporates microscale processes. Nat Commun.

[16] Moyano FE, Manzoni S, Chenu C (2013). Responses of soil heterotrophic respiration to moisture availability: an exploration of processes and models. Soil Biol Biochem.

[17] Huang W, Han T, Liu J, Wang G, Zhou G, Niu S (2016). Changes in soil respiration components and their specific respiration along three successional forests in the subtropics. Funct Ecol.

[18] Wei H, Chen X, Xiao G, Guenet B, Vicca S, Shen W (2015). Are variations in heterotrophic soil respiration related to changes in substrate availability and microbial biomass carbon in the subtropical forests?. Sci Rep.

[19] Lloyd J, Taylor JA. On the temperature dependence of soil respiration. Funct Ecol. 1994:315–23.

[20] Chen X, Deng Q, Lin G, Lin M, Wei H (2018). Changing rainfall frequency affects soil organic carbon concentrations by altering non-labile soil organic carbon concentrations in a tropical monsoon forest. Sci Total Environ.

[21] Chen XM, Li YL, Mo JM, Otieno D, Tenhunen J, Yan JH (2012). Effects of nitrogen deposition on soil organic carbon fractions in the subtropical forest ecosystems of Southern China. J Plant Nutr Soil Sci.

[22] Barreto MSC, Ramlogan M, Oliveira DMS, Verburg EEJ, Elzinga EJ, Rouff AA (2021). Thermal stability of soil organic carbon after long-term manure application across land uses and tillage systems in an oxisol. Catena.

[23] Peltre C, Fernández JM, Craine JM, Plante AF (2013). Relationships between biological and thermal indices of soil organic matter stability differ with soil organic carbon level. Soil Sci Soc Am J.

[24] Lee H, Rahn T, Throop H (2012). An accounting of C-based trace gas release during abiotic plant litter degradation. Global Change Biol.

[25] Hall SJ, Silver WL (2013). Iron oxidation stimulates organic matter decomposition in humid tropical forest soils. Glob Chang Biol.

[26] Bhattacharyya A, Campbell AN, Tfaily MM, Lin Y, Kukkadapu RK, Silver WL (2018). Redox fluctuations control the coupled cycling of iron and carbon in tropical forest soils. Environ Sci Technol.

[27] Jones ME, LaCroix RE, Zeigler J, Ying SC, Nico PS, Keiluweit M (2020). Enzymes, manganese, or iron? Drivers of oxidative organic matter decomposition in soils. Environ Sci Technol.

[28] Li D, Mo J, Fang Y, Xue J (2004). Study on availability of micronutrients in soils under three different forests of Dinghushan Nuture Reserve. Guihaia.

[29] Li Y, Shahbaz M, Zhu Z, Deng Y, Tong Y, Chen L (2021). Oxygen availability determines key regulators in soil organic carbon mineralisation in paddy soils. Soil Biol Biochem.

[30] Davidson EA, Janssens IA (2006). Temperature sensitivity of soil carbon decomposition and feedbacks to climate change. Nature.

[31] Zhao Q, Dunham-Cheatham S, Adhikari D, Chen C, Patel A, Poulson SR (2020). Oxidation of soil organic carbon during an anoxic-oxic transition. Geoderma.

[32] Barcellos D, O’Connell C, Silver W, Meile C, Thompson A (2018). Hot spots and hot moments of soil moisture explain fluctuations in iron and carbon cycling in a humid tropical forest soil. Soil Systems.

[33] Boone RD, Nadelhoffer KJ, Canary JD, Kaye JP (1998). Roots exert a strong influence on the temperature sensitivity of soil respiration. Nature.

[34] Wei H, Guenet B, Vicca S, Nunan N, AbdElgawad H, Pouteau V (2014). Thermal acclimation of organic matter decomposition in an artificial forest soil is related to shifts in microbial community structure. Soil Biol Biochem.

[35] Zhou G, Wei X, Wu Y, Liu S, Huang Y, Yan J (2011). Quantifying the hydrological responses to climate change in an intact forested small watershed in Southern China. Global Change Biol.

[36] Piao S, Fang J, Ciais P, Peylin P, Huang Y, Sitch S (2009). The carbon balance of terrestrial ecosystems in China. Nature.

[37] Nottingham AT, Meir P, Velasquez E, Turner BL (2020). Soil carbon loss by experimental warming in a tropical forest. Nature.

[38] Blagodatskaya Е, Zheng X, Blagodatsky S, Wiegl R, Dannenmann M, Butterbach-Bahl K (2014). Oxygen and substrate availability interactively control the temperature sensitivity of CO_2_ and N_2_O emission from soil. Biol Fert Soils.

[39] Walz J, Knoblauch C, Böhme L, Pfeiffer E-M (2017). Regulation of soil organic matter decomposition in permafrost-affected Siberian tundra soils - Impact of oxygen availability, freezing and thawing, temperature, and labile organic matter. Soil Biol Biochem.

[40] Wagg C, Schlaeppi K, Banerjee S, Kuramae EE, van der Heijden MGA (2019). Fungal-bacterial diversity and microbiome complexity predict ecosystem functioning. Nat Commun.

[41] Benton TG, Solan M, Travis JM, Sait SM (2007). Microcosm experiments can inform global ecological problems. Trends Ecol Evol.

[42] Schjønning P (1991). Soil mechanical properties of seven Danish Soils..

[43] Ward CP, Nalven SG, Crump BC, Kling GW, Cory RM (2017). Photochemical alteration of organic carbon draining permafrost soils shifts microbial metabolic pathways and stimulates respiration. Nat Commun.

[44] Zhou G, Guan L, Wei X, Zhang D, Zhang Q, Yan J (2006). Litterfall production along successional and altitudinal gradients of subtropical monsoon evergreen broadleaved forests in Guangdong, China. Plant Ecol.

[45] Tang X, Liu S, Zhou G, Zhang D, Zhou C (2006). Soil-atmospheric exchange of CO_2_, CH_4_, and N_2_O in three subtropical forest ecosystems in southern China. Global Change Biol.

[46] Qin SQ, Chen LY, Fang K, Zhang QW, Wang J, Liu FT (2019). Temperature sensitivity of SOM decomposition governed by aggregate protection and microbial communities. Sci Adv.

